# Bone Healing Response to Different Concentrations of Nano-Hydroxyapatite Incorporated into Mineral Trioxide Aggregate and Bioceramic Sealers

**DOI:** 10.3390/polym18141743

**Published:** 2026-07-16

**Authors:** Arkhawan Ali Abdulhaq, Chenar Anwar Mohammad, Bassam Karem Amin

**Affiliations:** College of Dentistry, Hawler Medical University, Erbil 44001, Iraq; arkhawan.ali@hmu.edu.krd (A.A.A.); chenar.anwar@hmu.edu.krd (C.A.M.)

**Keywords:** nano-hydroxyapatite, bone regeneration, histomorphometry, biomaterials, calcium silicate cement, rabbit model

## Abstract

Nano-engineering strategies have been increasingly applied to enhance the biological performance of calcium silicate-based materials; however, the optimal concentration of nano-hydroxyapatite (HANP) remains unclear. This study evaluated the bone-healing response to different concentrations of HANP incorporated into mineral trioxide aggregate (MTA) and bioceramic (BC) sealers in an experimental rabbit model. Thirty adult New Zealand white rabbits were allocated into two experimental groups according to sealer type: HANP-modified MTA and HANP-modified BC (*n* = 15 each). Two standardized circular intrabony defects were created bilaterally in the maxillary diastema of each rabbit. In the MTA group, the right-side defects were filled with 2% and 4% HANP-modified MTA, while the left-side defect received 6% HANP-modified MTA, and the adjacent defect was left untreated as a control. The same protocol was followed for the BC group using corresponding HANP concentrations. Five rabbits per group were sacrificed at 2, 4, and 8 weeks postoperatively for histopathological hematoxylin and eosin (H&E) and Masson trichrome staining. The results demonstrated significant differences among groups at all time points, with 4% HANP showing the most favorable biological response, including reduced inflammatory cell infiltration, increased new bone formation, and improved collagen organization compared with lower and higher concentrations. Pairwise comparisons at matched HANP concentrations revealed no statistically significant differences between HANP-modified MTA and BC groups. These findings indicate that HANP incorporation enhances the biological performance of calcium silicate-based sealers in a concentration-dependent manner, with 4% representing the most favorable response among the concentrations evaluated for promoting bone regeneration.

## 1. Introduction

A bone defect is characterized by the absence of bone tissue in a particular body component that may develop as a result of trauma, infection, inflammation, injury, or congenital conditions [1]. In certain instances, it may also result from persistent medical conditions or surgical operations [2]. To promote bone regeneration, materials must frequently be surgically implanted [3,4]. In recent decades, autograft materials have largely been used for bone repair because of their superior osteogenesis, inducibility, and bone conduction [5]. In clinical practice, autologous bone grafts remain the preferred treatment for bone repair [6]. Nevertheless, issues with the harvesting process and the scarcity of donor bone restrict this technique’s practical use [7]. Because of this, alternate graft materials and other bone replacements are essential for successful bone restorations [8].

An ideal bone substitute should meet requirements including bioresorbability, osteoinduction, and osteoconductivity [9]. Additionally, it should be thermally nonconductive and immune rejection-free [10]. Many alloplastic materials have been thoroughly investigated for use as novel bone substitutes, including hydroxyapatite, tri-calcium phosphate, calcium phosphate cement, calcium sulphate, bone morphogenetic protein, ceramic, and ceramic composites [11]. Nano-hydroxyapatite-based biomaterials were able to promote the bone repair process without any signs of inflammation. Since the composition of hydroxyapatite is similar to that of bone minerals, it was anticipated that HA would provide a suitable surface for cell adhesion [12] and because of the lack of protein, it does not cause allergic and immune reactions [13]. Furthermore, the release of calcium ions from the nanocrystalline HA is faster than in coarser crystals. Thus, bioceramics based on nanosized HA are a material of choice for various biomedical applications because of improved mechanical properties, sinterability, densification, cellular attachment, proliferation, and differentiation [14]. The nanostructure of HA also facilitates the adsorption of proteins and growth factors, further promoting the osteoinduction process, where new bone tissue is formed at the implant site [15].

The bioactivity of MTA is attributed to its ability to promote tissue mineralization, cell migration, and proliferation. Calcium silicate, the main ingredient of MTA, changes into calcium hydroxide when it comes into contact with tissue. Calcium hydroxide then breaks down into calcium and hydroxide ions, continuously releasing them into the environment [16]. Because of its composition, which enables cells to adhere and proliferate quickly on its structure, it can aid in bone healing. With direct bone apposition, there is evidence that MTA encourages a positive reaction in the osseous environment. MTA surfaces promote RunX2 expression, matrix formation, and osteoblast cell attachment, all of which are critical for osteogenesis [17].

The newer generation of bioceramic sealers has been developed to show the potential of lasting bioactivity by the diffusion of molecules during and after their setting, and may modulate the apical tissue environment through either direct contact of these molecules with the apical tissues, as in cases of extrusion, or indirectly by diffusion from the root canal system. After the removal of bacteria, healing of apical periodontitis requires remodeling of the granulomatous tissue and induction of the proliferation of bone marrow stem cells and osteoblast precursors into mature osteoblasts, leading to remineralization of the apical tissues [18].

The biological threshold at which nanoparticle incorporation enhances osteogenesis without compromising tissue compatibility remains unclear. Although nano-engineering approaches have shown promise in improving the bioactivity of calcium silicate-based materials, there is a lack of comparable in vivo studies evaluating different nanoparticle concentrations across various sealer matrices. Addressing this gap is essential for translating nanotechnology-based modifications into clinically relevant regenerative outcomes. Therefore, the present study aimed to evaluate the concentration-dependent bone healing response of mineral trioxide aggregate (MTA) and bioceramic (BC) sealers modified with varying concentrations of nano-hydroxyapatite using histological analysis and Masson’s trichrome staining, in an experimental rabbit model. The null hypothesis was that varying concentrations of nano-hydroxyapatite would not significantly affect inflammatory response, new bone formation, or collagen deposition, irrespective of sealer type.

## 2. Materials and Methods

### 2.1. Materials

Nano-hydroxyapatite (MERK, Darmstadt, Germany), with particle sizes ranging from approximately 20.98 to 39.55 nm as determined by SEM analysis, were incorporated into mineral trioxide aggregate (MTA) (MTA Cem, NEXOBIO Co., Cheongju-si, Republic of Korea) and a bioceramic sealer (BC) (ONE-FIL bioceramic sealer, MEDICLUS Co., Ltd., Cheongju-si, Republic of Korea) at concentrations of 2%, 4%, and 6% (*w*/*w*), according to a protocol previously described in an in vitro study [19].

### 2.2. Study Design

The study was conducted in accordance with ARRIVE guidelines for preclinical in vivo studies (NC3Rs, London, UK) and was approved by the Research Ethics Committee of the College of Dentistry, Hawler Medical University, Erbil, Kurdistan Region, Iraq. All procedures adhered to ethical guidelines for the humane treatment of animals, including appropriate housing, medical care, and anesthesia protocols. The study objectives could not be achieved through non-animal models, and the minimum necessary number of animals was used to ensure statistical validity. The required sample size for this experimental animal study was determined by using G*Power software (version 3.1.9.2; Düsseldorf, Germany). The statistical parameters were set at a power of 80% (1 − β = 0.80) and a significance level of α = 0.05, with an estimated effect size (f) of 0.40 and four experimental conditions (2%, 4%, and 6% HANP-modified sealer and control), corresponding to three degrees of freedom. Power analysis indicated a sample size equivalent to 60 bone defects per material group. Under the split-mouth design, each rabbit contributed four standardized intrabony defects; therefore, 15 rabbits were required per material group, resulting in a total of 30 rabbits to detect statistically significant differences between experimental groups.

### 2.3. Animal Selection and Housing

**Figure 1:**
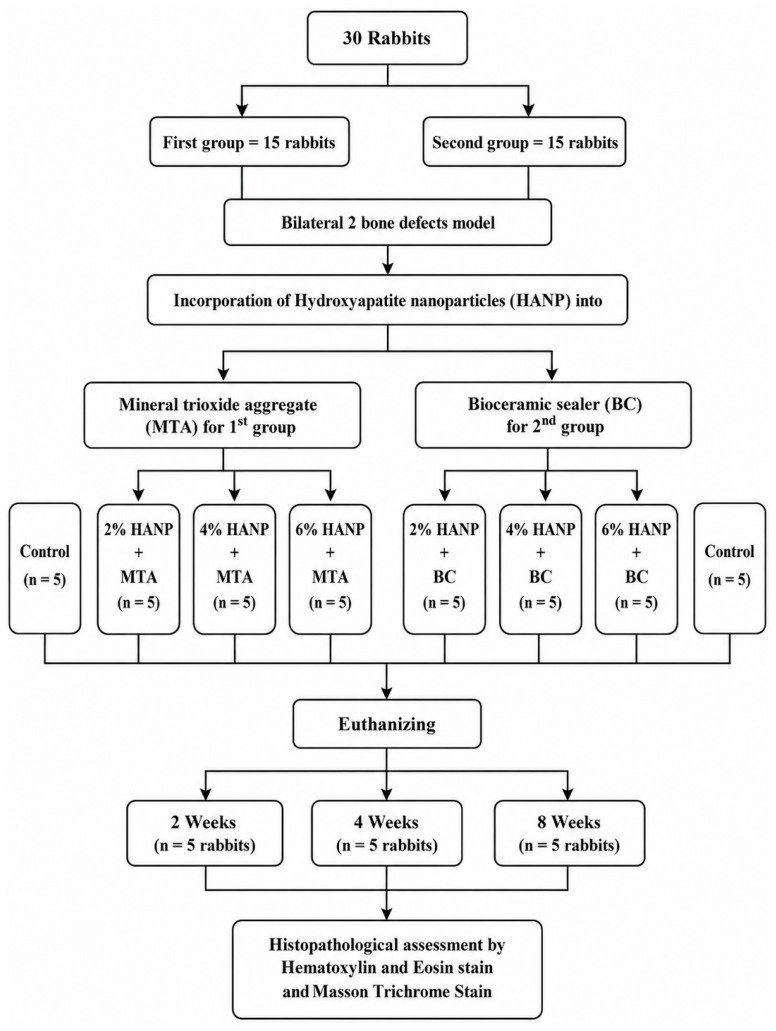
Flowchart showing the experimental stages and their order fulfillment.

A total of 30 adult male New Zealand white rabbits, aged 6–8 months and weighing 1.5–2 kg, were included in this study, in which 120 maxillary bone defects were created. Animals were housed at the Central Animal Care Facility, College of Pharmacy, Hawler Medical University, Erbil. They were acclimatized for one week before surgery and maintained under standardized environmental conditions (temperature: 19–21 °C; humidity: 45% ± 10%; light/dark cycle: 12:12 h), with free access to water and a standard diet ad libitum. Fifteen rabbits (60 defects) were assigned to the HANP-modified MTA group, and fifteen rabbits (60 defects) were assigned to the HANP-modified BC group. In each rabbit, four standardized intrabony defects (3 mm in diameter and 4 mm in depth) were created on the right and left buccal aspects of the maxillary diastema, according to a previously described protocol [20]. A split-mouth design was used in which the two right-sided defects were assigned to receive 2% and 4% HANP-modified MTA, respectively, whereas the first left-sided defect was filled with 6% HANP-modified MTA, and the adjacent defect was left untreated as a control. In the BC group, the same protocol was followed, except that 2%, 4%, and 6% HANP were incorporated into the BC sealer [21]. Five rabbits from each group were euthanized at 2, 4, and 8 weeks postoperatively, and the maxillae containing the defect sites were harvested for histopathological analysis, as shown in (Figure 1).

### 2.4. Surgical Protocol

**Figure 2:**
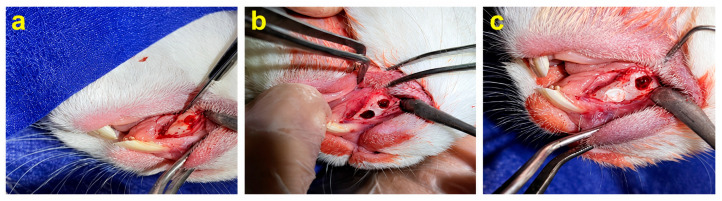
Surgical creation of standardized bone defects in the rabbit maxilla. (**a**) Exposure of the diastema region following reflection of a full-thickness mucoperiosteal flap. (**b**) Preparation of the buccal maxillary surface and creation of standardized circular bone defects using a low-speed round bur under continuous sterile saline irrigation. (**c**) Placement of experimental materials within the defects according to group allocation; control defects were left unfilled.

General anesthesia was induced by intramuscular administration of ketamine hydrochloride (20 mg/kg body weight) and xylazine (0.2 mL/kg body weight, 2%) before surgery. In addition, oxytetracycline (20%) was administered intramuscularly at 1 mL/kg body weight, one hour before surgery, and postoperative antibiotic prophylaxis was continued at the same dose for 7 days. To prevent corneal drying during anesthesia, tetracycline ophthalmic ointment was applied. Two surgical incisions were made on each buccal aspect of the natural edentulous diastema, located between the central incisors and premolars, to raise a mucoperiosteal flap (Figure 2a–c). Two standardized intrabony defects were created in each diastema using intermittent drilling with a round bur (size #010), followed by a fissure bur (size #010), under copious irrigation, according to a previously described protocol [20]. Experimental materials were inserted into each defect until completely filled. Flaps were repositioned and sutured using absorbable braided polyglycolic acid 6-0 sutures (Yingmed, Ningbo, China). Five rabbits from each group were euthanized by an overdose of ketamine hydrochloride at 2, 4, and 8 weeks postoperatively. The right and left maxillary segments containing the defect sites and surrounding bone were harvested for histopathological analysis.

### 2.5. Histopathological Assessment

At each predetermined healing interval, defect sites were harvested and fixed by immersion in 10% neutral buffered formalin for 48–72 h, and subsequently decalcified in 10% formic acid for approximately 2–3 days. Following decalcification, the specimens were dehydrated through graded ethanol solutions, cleared in xylene, and embedded in paraffin wax for histological processing. Serial sections approximately 5 µm thick were obtained through the center of each defect using a rotary microtome (Leica RM2135, Leica Microsystems, Wetzlar, Germany) [22]. Representative photomicrographs were captured, and histopathological examination was performed by an experienced histopathologist who was blinded to the experimental groups to reduce observer bias. All slides were assessed at ×40, ×100, and ×400 magnifications [23].

### 2.6. Histomorphometric Assessment

Quantitative histomorphometric evaluation of bone regeneration within the defect area was performed using a digital light microscope connected to an image analysis system (Motic/ToupTek, ToupView (×86), version 3.7.4183, 2014) at magnifications of ×40, ×100, and ×400. Digital photomicrographs of microscopic fields covering the entire defect region were captured for analysis.

For quantitative analysis, five random microscopic fields per section were evaluated for each animal. All assessments were performed in a blinded manner, and the mean values of the five fields were calculated [24].

The quantity of inflammatory cells was assessed using a semi-quantitative four-point ordinal scale based on the number of inflammatory cells observed per ×400 field as follows: low: no inflammatory cells or extremely few (less than five cells), mild (5 to <25 cells), moderate (25–125 cells), and severe (>125 cells) [25].

New bone formation was assessed using a semi-quantitative histological scoring system. The amount of newly formed bone within the defect area was evaluated under light microscopy and graded as follows. Score 0: Absence of new bone tissue formation. Score 1: Discrete. Small bone trabeculae covering <25% of the defect area. Score 2: Moderate. New bone tissue formation covering 25–50% of the defect area. Score 3: Extensive. Complete coverage with formation of a bony bridge around the biomaterial tested [26].

### 2.7. Special Stains

Perl’s Prussian blue stain was employed to identify iron deposition within the bone trabeculae. The quantity and arrangement of collagen fibers were also evaluated using Masson trichrome stain (HistoPlus, İzmir, Turkey). The following semi-quantitative method was used to assess the degree of collagen organization and maturation [27]. Score 0: Early, immature collagen fibers organized in a reticular pattern are present. Score 1: A moderate number of collagen fibers that form weakly cohesive bundles are present. Score 2: Mature collagen fibers arranged into compact bundles are predominantly present.

### 2.8. Masson’s Trichrome Staining Protocol

After the specimens were fixed and decalcified, paraffin-embedded slices (5 µm) were prepared. Sections underwent xylene deparaffinization, graded ethanol rehydration, and distilled water washing in preparation for Masson’s Trichrome staining. After applying Bouin’s solution for one hour at 56 °C to improve staining, the sections were cooled and rinsed under running water. Biebrich scarlet-acid fuchsin was used for 5 min after slices were rinsed and nuclei were stained for 10 min with Weigert’s iron hematoxylin. Aniline blue was used to stain collagen fibers for five minutes after differentiation was accomplished using phosphomolybdic-phosphotungstic acid solution. This allowed for the evaluation of collagen deposition and new bone development in the defect fields [28,29].

### 2.9. Statistical Analysis

Statistical analysis was performed using SPSS version 27 (IBM Corp., Armonk, NY, USA). Data were assessed for normality using the Shapiro–Wilk test. As the data were not normally distributed, results were expressed as median and interquartile range (IQR), while mean ± standard deviation (SD) was also reported for descriptive purposes. Intergroup comparisons were performed using the Kruskal–Wallis test, followed by Bonferroni-adjusted post hoc pairwise comparisons. A *p*-value < 0.05 was considered statistically significant. Graphs were generated using GraphPad Prism version 10 (GraphPad Software Inc., San Diego, CA, USA).

## 3. Results

### 3.1. Inflammatory Response

The median (IQR) values, together with mean ± standard deviation (SD), of inflammatory response around the implanted materials at 2, 4, and 8 weeks are presented in Table 1. The results demonstrated a higher inflammatory response in the 2% and 6% HANP-containing groups, particularly during the early healing phase (2 weeks), followed by a gradual reduction at later time points. In contrast, defects treated with 4% HANP formulations exhibited the lowest inflammatory response, with a progressive decrease in inflammatory cell infiltration from 2 to 8 weeks in both 4% HANP + MTA and 4% HANP + BC groups.

**Figure 3:**
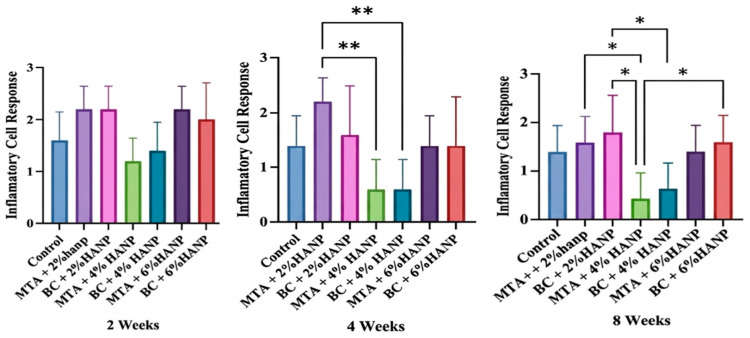
Pairwise comparison for inflammatory cell response among untreated empty control defects, MTA, and BC sealer-modified with 2%, 4%, and 6% HANP at 2 Weeks, 4 Weeks, and 8 Weeks intervals. * *p* < 0.05; ** *p* < 0.01.

For pairwise comparison of inflammatory cell response among different groups using Bonferroni-adjusted pairwise comparisons test, (Figure 3), shows that 4% HANP + MTA group at 2 weeks, exhibited significantly lower inflammatory scores compared to 6% HANP + BC, 6% HANP + MTA, 2% HANP + MTA, and 2% HANP + BC. Similarly, the 4% HANP + BC group demonstrated significantly reduced inflammatory reaction compared with 6% HANP + MTA, 2% HANP + MTA, and 2% HANP + BC. However, no significant differences were found between both 4% HANP groups (MTA and BC) at 2 weeks (*p* = 0.583).

At 4 weeks, 4% HANP groups (MTA and BC) showed significantly lower inflammatory cell response compared to 2% HANP + MTA groups, with no significant differences detected between both 4% HANP groups (MTA & BC) at 4 weeks (*p* = 1.000). At 8 weeks, the 4% HANP + MTA group demonstrated significantly reduced inflammatory response compared to the control, 6% HANP + BC, 6% HANP + MTA, 2% HANP + MTA, and 2% HANP + BC groups. Likewise, the 4% HANP + BC group showed significantly lower inflammatory scores compared to 6% HANP + BC, 2% HANP + MTA, and 2% HANP + BC groups. However, non-significant differences were observed between both 4% HANP groups (*p* = 0.712) and among the remaining studied groups comparisons (*p* > 0.05).

### 3.2. New Bone Formation

The median (IQR) values, together with mean ± standard deviation (SD), of new bone formation around the experimental materials after 2 weeks, 4 weeks and 8 weeks follow up are presented in Table 2, and show that the highest new bone formation at all time points was observed in the 4% HANP groups, particularly 4% HANP + MTA, followed by 4% HANP + BC, then the 6% HANP groups, whereas control and 2% HANP + BC exhibited the lowest levels of new bone formation.

**Figure 4:**
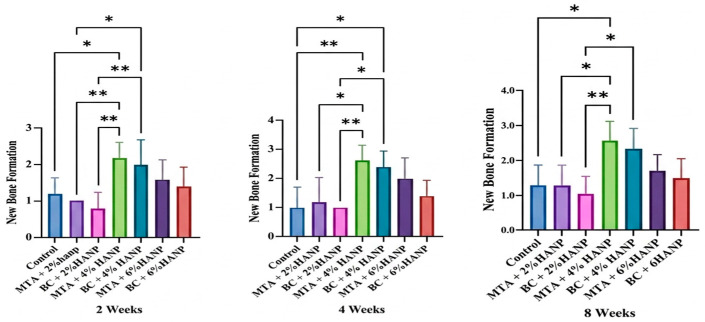
Pairwise comparison for new bone formation among untreated empty control defects, MTA, and BC sealer-modified with 2%, 4%, and 6% HANP at 2 Weeks, 4 Weeks, and 8 Weeks intervals. * *p* < 0.05; ** *p* < 0.01.

For pairwise comparisons among the different studied groups using the Bonferroni test, (Figure 4), shows significant variations in new bone formation among different studied groups. At 2 weeks, the 2% HANP + BC group exhibited significantly lower new bone formation compared with 6% HANP + MTA, 4% HANP + BC, and 4% HANP + MTA groups, while the 4% HANP + MTA group demonstrated significantly greater bone formation compared to the control and 2% HANP + MTA group.

At 4 weeks, significantly higher bone formation was observed in both 4% HANP groups (MTA and BC) with non-significant differences between the two groups (*p* = 0.755). Additionally, the 4% HANP + MTA group showed significantly greater bone formation compared to the control, 2% HANP + BC, 2% HANP + MTA, and 6% HANP + BC groups.

**Figure 5:**
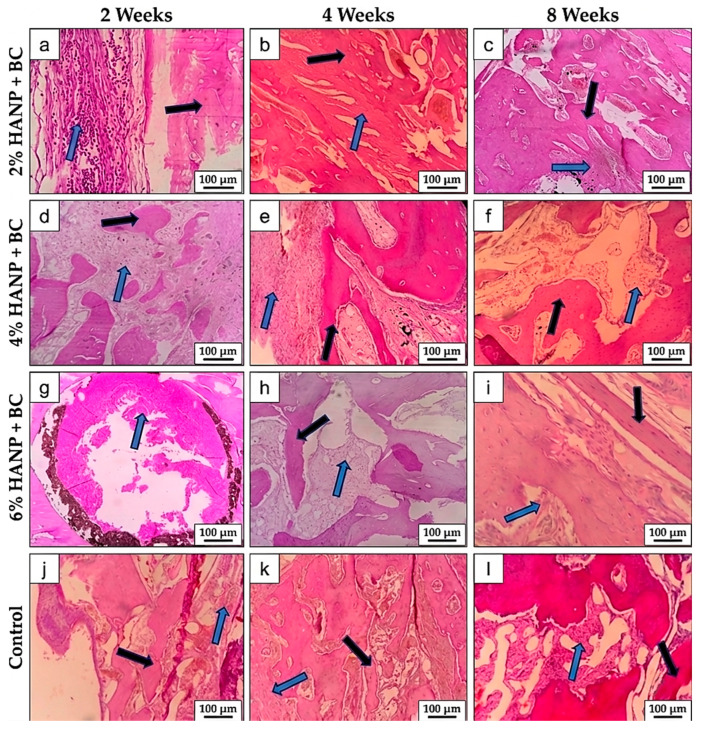
Representative hematoxylin and eosin (H&E)-stained histological sections showing bone regeneration and inflammatory response in defects treated with bioceramic (BC) sealer containing nano- hydroxyapatite (HANP). Panels (**a**–**c**) 2% HANP + BC; (**d**–**f**) 4% HANP + BC; (**g**–**i**) 6% HANP + BC; and (**j**–**l**) untreated empty control defects, evaluated at 2, 4, and 8 weeks. Black arrows indicate newly formed trabecular bone, and blue arrows indicate inflammatory cell infiltration. Magnification ×100.

**Figure 6:**
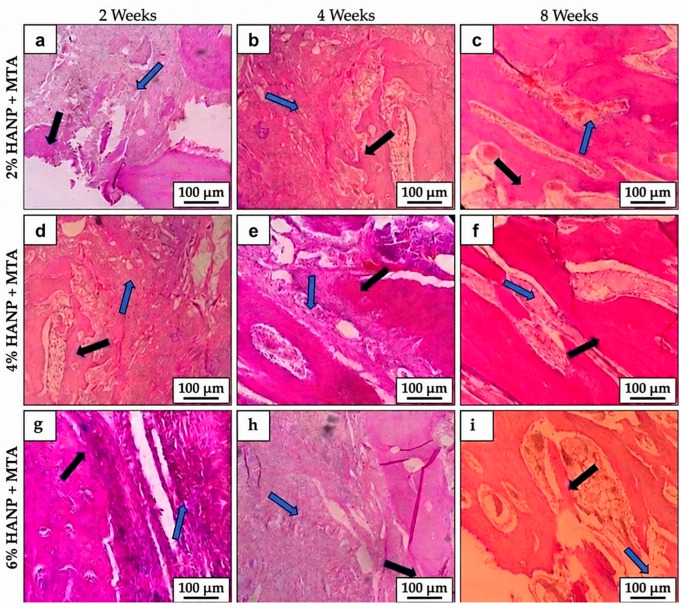
Representative hematoxylin and eosin (H&E)-stained histological sections showing bone regeneration and inflammatory response in defects treated with mineral trioxide aggregate (MTA) containing nano-hydroxyapatite (HANP). Panels (**a**–**c**) 2% HANP + MTA; (**d**–**f**) 4% HANP + MTA; and (**g**–**i**) 6% HANP + MTA, evaluated at 2, 4, and 8 weeks. Black arrows indicate newly formed trabecular bone, and blue arrows indicate inflammatory cell infiltration. Magnification ×100.

At 8 weeks, 4% HANP + MTA and 4% HANP + BC groups demonstrated significantly higher bone formation than 2% HANP + BC group, with non-significant differences between both 4% HANP + MTA and 4% HANP + BC groups (*p* = 0.710). Furthermore, the 4% HANP + MTA group continued to show significantly more new bone formation compared to the control, 2% HANP + MTA, and 6% HANP + BC groups. These quantitative findings were consistent with the histopathological observations, where sections from the 4% HANP groups demonstrated reduced inflammatory cell infiltration and more organized tissue architecture compared with the other experimental groups (Figure 5 and Figure 6).

### 3.3. Masson Trichrome Staining Results

**Figure 7:**
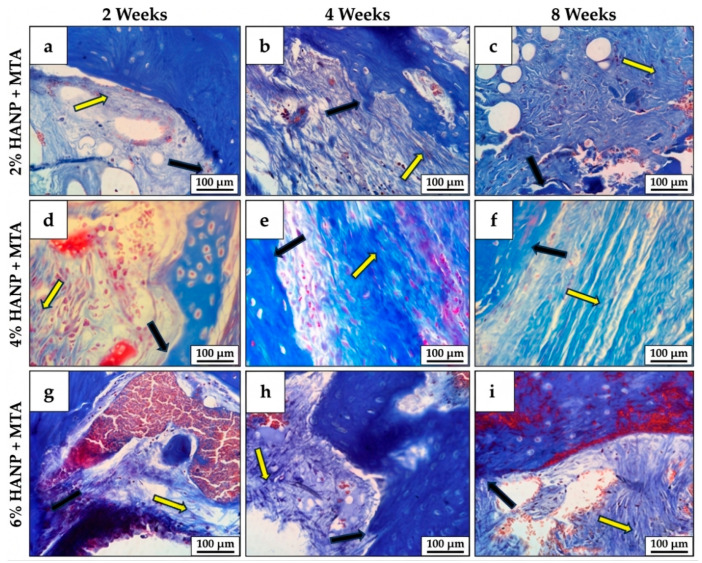
Representative Masson’s trichrome-stained histological sections showing bone regeneration and collagen deposition in defects treated with mineral trioxide aggregate (MTA) containing nano- hydroxyapatite (HANP). Panels (**a**–**c**) show 2% HANP + MTA, (**d**–**f**) 4% HANP + MTA, and (**g**–**i**) 6% HANP + MTA at 2, 4, and 8 weeks. Black arrows indicate newly formed bone trabeculae, and yellow arrows indicate collagen deposition. Magnification ×400.

**Figure 8:**
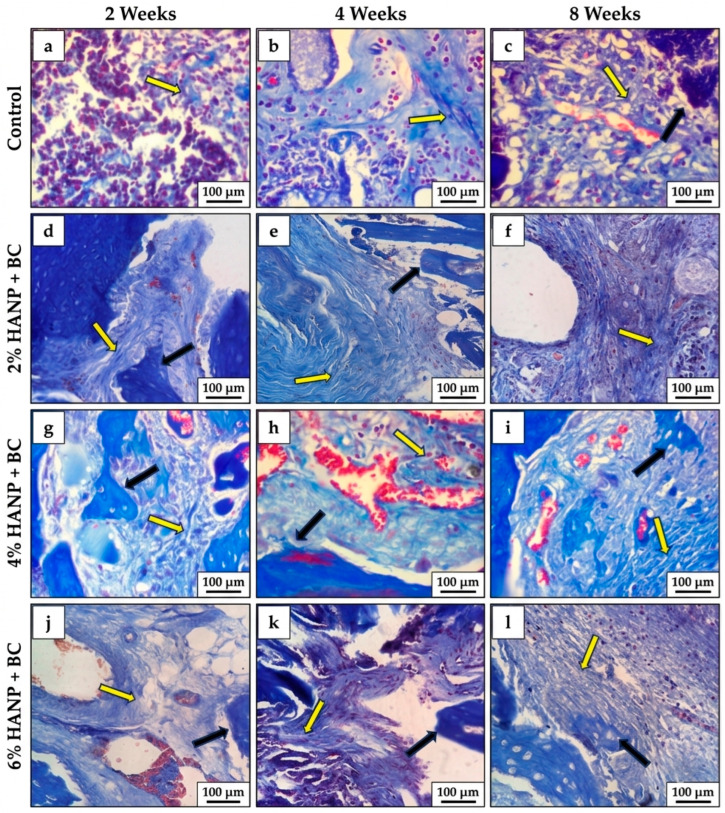
Representative Masson’s trichrome-stained histological sections showing bone regeneration and collagen deposition in control defects and defects treated with bioceramic (BC) sealer containing nano- hydroxyapatite (HANP). Panels (**a**–**c**) show untreated empty control defects, (**d**–**f**) 2% HANP + BC, (**g**–**i**) 4% HANP + BC, and (**j**–**l**) 6% HANP + BC at 2, 4, and 8 weeks. Black arrows indicate newly formed bone trabeculae, and yellow arrows indicate collagen deposition. Magnification ×400.

The results show a progressive increase in collagen fiber deposition and maturation over time in all experimental groups (Figure 7 and Figure 8). At two weeks, the control group predominantly exhibited early immature collagen fibers arranged in a reticular pattern. Limited or low collagen deposition was observed in the 2% HANP groups, whereas the 4% HANP groups showed more organized collagen. In contrast, the 6% HANP + MTA group demonstrated minimal collagen deposition, while the 6% HANP + BC group presented mild collagen fiber formation.

At four weeks, collagen deposition increased in most groups, with moderate collagen bundles becoming more evident within the defect area. The 4% HANP groups demonstrated more pronounced collagen bundle formation compared with the other groups, whereas the control group continued to show only mild collagen fiber deposition. At eight weeks, further maturation of collagen fibers was observed, with several experimental groups demonstrating predominantly compact collagen bundles. The 4% HANP + MTA group exhibited the most advanced collagen organization, followed by the 4% HANP + BC group, while the 2% and 6% HANP groups showed moderate collagen maturation. The median (IQR) values, together with mean ± standard deviation (SD), of collagen fiber deposition and maturation around the implanted materials at the respective time intervals are presented in Table 3.

**Figure 9:**
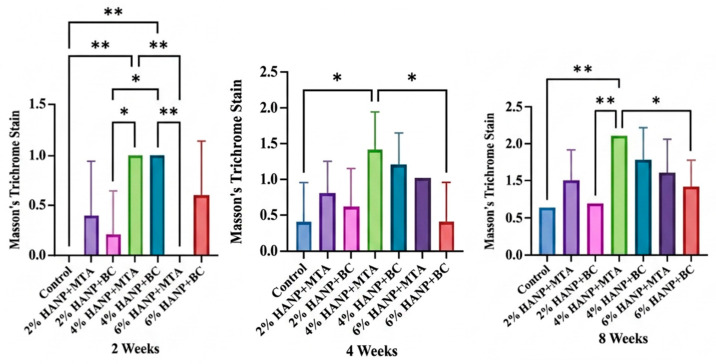
Pairwise comparison for Masson’s trichrome stain of control untreated empty defects, MTA, and BC sealer-modified with 2%, 4%, and 6% HANP at 2 Weeks, 4 Weeks, and 8 Weeks intervals. * *p* < 0.05; ** *p* < 0.01.

For pairwise comparison of collagen fiber deposition among different groups using the Bonferroni-adjusted pairwise comparison test. (Figure 9), shows that at 2 weeks, the 4% HANP + MTA group exhibited significantly higher collagen fiber deposition compared with the control and 2% HANP + BC groups. Similarly, the 4% HANP + BC group demonstrated significantly greater collagen fiber formation as compared with the control group. In addition, the 6% HANP groups showed variable responses, with 6% HANP + BC demonstrating moderate collagen deposition, whereas 6% HANP + MTA exhibited minimal collagen formation comparable to the control group. However, no significant differences were detected between the two 4% HANP groups (MTA and BC) at 2 weeks (*p* > 0.05). The 4% HANP + MTA group exhibited the highest mean staining score at 4 week’s intervals, indicating a greater degree of collagen maturation and extracellular matrix organization compared with the other groups. Statistical analysis showed that the 4% HANP + MTA group had significantly higher staining scores than the control group and the 6% HANP + BC group (*p* < 0.05). The 4% HANP + BC group also demonstrated relatively high collagen deposition, although its values were slightly lower than those of the 4% HANP + MTA group, and the difference between these two groups was not statistically significant. At 8 weeks, the 4% HANP + MTA group demonstrated significantly greater collagen deposition compared with the control and 6% HANP + BC groups. Likewise, the 4% HANP + BC group exhibited significantly higher collagen maturation compared with the control group. However, non-significant differences were detected between both 4% HANP groups and among the remaining studied group (*p* > 0.05).

## 4. Discussion

The present study demonstrated that the incorporation of nano-hydroxyapatite (HANP) into mineral trioxide aggregate (MTA) and bioceramic (BC) sealers significantly influences bone regeneration in a concentration-dependent manner. Among the tested formulations, the 4% HANP concentration provided the most favorable balance between enhanced new bone formation and reduced inflammatory response. Histological findings across all observation periods confirmed that both the material composition and nanoparticle concentration are critical determinants of in vivo tissue healing outcomes. Hydroxyapatite-based biomaterials are widely recognized for their osteoconductive properties and their ability to support bone regeneration. In particular, nano-hydroxyapatite (HANP) has gained increasing attention due to its high surface area and close resemblance to the mineral phase of natural bone, which enhances cellular interactions and promotes osteoblast activity and matrix mineralization [30]. Clinical and preclinical evidence further supports the use of nanocrystalline hydroxyapatite as an effective bone substitute, demonstrating improved bone regeneration outcomes comparable to conventional graft materials [31]. However, despite these advantages, the biological response to HANP is highly dependent on its physicochemical characteristics and concentration. Variations in nanoparticle size, structure, and surface properties can modulate inflammatory responses and cellular behavior, potentially leading to either enhanced healing or undesirable tissue reactions [32]. This highlights a critical gap in current knowledge regarding the optimal concentration of HANP, particularly when incorporated into bioactive endodontic materials.

Mineral trioxide aggregate (MTA) and bioceramic (BC) sealers are extensively used in endodontics due to their sealing ability and inherent bioactivity, including the capacity to induce hard tissue formation. Nevertheless, their regenerative performance can still be optimized, especially in terms of accelerating bone formation and modulating inflammation. The incorporation of HANP into these materials represents a promising strategy to enhance their osteogenic potential, as composite nano-hydroxyapatite-based systems have been shown to further stimulate bone regeneration compared to conventional materials alone [33]. Accordingly, the present study aimed to address the lack of consensus regarding the most effective HANP concentration by systematically evaluating its incorporation into MTA and BC sealers.

The rabbit model is widely utilized in craniofacial bone regeneration research, including maxillary and alveolar bone defect studies, due to its anatomical suitability and translational relevance to dental applications. Experimental models in rabbits allow the evaluation of bone substitutes in both cranial (non-load-bearing) and alveolar or mandibular (load-bearing) regions, which exhibit distinct healing characteristics relevant to maxillofacial reconstruction [34].

The present findings demonstrate that inflammatory cell infiltration is strongly influenced by both nano-hydroxyapatite (HANP) concentration and healing time, exhibiting clear concentration- and time-dependent trends. The elevated inflammatory response observed at 2 weeks across most groups reflects the expected acute inflammatory phase following biomaterial implantation, which is essential for initiating tissue repair and regeneration. This response is consistent with established evidence indicating that early leukocyte recruitment and macrophage activation are critical regulators of healing outcomes and biomaterial integration [32]. A progressive reduction in inflammation at 4 and 8 weeks further supports the pivotal role of time in the resolution of the inflammatory process, in agreement with previous reports [35].

The pronounced early inflammatory response may also be partially attributed to the intrinsic properties of the base materials. Calcium silicate-based sealers are characterized by a high alkaline pH, which can stimulate the release of pro-inflammatory cytokines, including IL-1 and IL-6, during the initial healing phase [36]. In addition, their relatively delayed setting time allows maintenance of an elevated pH (up to ~12) for an extended period, enhancing antimicrobial activity while potentially prolonging tissue irritation [37]. Thermal changes associated with the setting reaction may further contribute to inflammatory cell recruitment and cytokine release [38].

Notably, the 4% HANP groups exhibited a consistently reduced inflammatory response at all-time points, suggesting a concentration-dependent immunomodulatory effect. This phenomenon may be explained by the ability of nano-hydroxyapatite to regulate macrophage polarization. Macrophages play a central role in inflammation by transitioning between the pro-inflammatory M1 phenotype and the anti-inflammatory, reparative M2 phenotype. Evidence indicates that HANP promotes M2 polarization while suppressing M1 activity [39]. Therefore, the 4% concentration may have established a microenvironment favoring M2 dominance, thereby facilitating resolution of inflammation and progression toward tissue regeneration. In contrast, both lower (2%) and higher (6%) concentrations appear less effective in achieving this balance, resulting in relatively increased inflammatory responses.

The comparatively improved performance of the 4% HANPs-modified groups may be explained that this concentration may provide a favorable balance between bioactivity and biocompatibility. This observation is consistent with previous experimental and clinical studies indicating that nano- hydroxyapatite (HANP) may modulate key inflammatory mediators, including interleukin-1β (IL-1β) and tumor necrosis factor-α (TNF-α), thereby reducing inflammatory burden and supporting improved healing outcomes [40].

In contrast, the increased inflammatory response observed in the 2% HANP-modified groups could be attributed to that this concentration is insufficient to induce effective bioactive and immunomodulatory effects. At low nanoparticle loading, the release of calcium and phosphate ions may not reach the threshold required to regulate macrophage behavior or promote the transition from an M1 to an M2 phenotype, leading to sustained inflammatory cell infiltration. Additionally, limited HANP incorporation may inadequately modify surface properties and sealing ability, thereby impairing protein adsorption, cell adhesion, and interfacial stability—factors essential for early inflammation resolution. This interpretation aligns with previous findings that the biological performance of nano-hydroxyapatite is highly concentration-dependent, with suboptimal concentrations failing to enhance bioactivity or modulate inflammation effectively [41].

Conversely, higher nanoparticle loading (6% HANP-modified groups) may introduce an excessive particulate burden that exacerbates early inflammatory responses. This may be attributed to increased macrophage activation and elevated expression of pro-inflammatory cytokines, resulting in prolonged inflammation and delayed healing [42].

The absence of significant differences between MTA- and BC-based materials within the 4% HANP groups further suggests that nanoparticle concentration plays a more critical role than the base material in determining the inflammatory outcome. This interpretation is supported by evidence indicating that calcium silicate-based sealers, including both MTA and bioceramic formulations, exhibit broadly comparable biocompatibility and tend to show reduced inflammatory responses over time, with differences often being material-specific but not always statistically significant [43].

The present study employed nano-hydroxyapatite (HANP) due to its well-documented osteoconductive properties and ability to enhance early bone remodeling. Previous studies have demonstrated that HANP promotes significantly greater bone formation compared to conventional biomaterials such as standard hydroxyapatite, tricalcium phosphate, and gelatin-based scaffolds [44,45,46]. This enhanced osteogenic potential has been partly attributed to the structural characteristics of HANP, particularly its porosity, which plays a critical role in both early and late stages of osteogenesis by facilitating cellular infiltration and vascularization, as proposed by Habibovic et al. [47]. Supporting evidence from both animal and human studies further indicates that pore size and interconnectivity significantly influence bone regeneration by promoting angiogenesis and tissue ingrowth [48].

In the present study, new bone formation increased progressively over time, demonstrating a clear concentration-dependent pattern influenced by both HANP content and material type. Among all groups, the 4% HANP formulation exhibited superior outcomes, characterized by enhanced trabecular organization, denser mineralized tissue deposition, and more continuous defect bridging compared with both lower (2%) and higher (6%) concentrations. These findings are consistent with previous evidence indicating that optimally dosed nano-hydroxyapatite enhances osteoblast adhesion, proliferation, and mineralization due to their nanoscale topography and chemical similarity to native bone mineral [49]. In contrast, lower concentrations may provide insufficient surface area for effective cell–material interaction, while higher concentrations may adversely affect material porosity and setting characteristics, thereby compromising biological performance [50].

The enhanced osteogenesis observed in the 4% HANP groups may also be attributed to increased alkaline phosphatase activity, which plays a key role in matrix mineralization. HANP provides a favorable scaffold for osteoprogenitor cell attachment and differentiation, thereby promoting early osteoid deposition and subsequent maturation of bone tissue [51]. Additionally, the nanoscale architecture and interconnectivity of HANP may facilitate angiogenesis and cell migration even in the absence of exogenous growth factors, further supporting defect repair and bone regeneration [52]. Collectively, these mechanisms may explain the superior osteogenic performance observed at the 4% concentration. In contrast, the control group demonstrated only moderate increases in bone formation over time, reflecting the limited regenerative capacity of untreated bone defects, as reported in previous animal studies [53].

The present findings are in agreement with prior in vivo studies demonstrating that HANP incorporation enhances early recruitment and differentiation of osteoprogenitor cells, leading to accelerated matrix deposition and maturation within defect sites [54]. The significant intergroup differences observed at later time points further confirm that HANP incorporation improves defect bridging, osteointegration, and overall bone regeneration without inducing adverse inflammatory responses [55]. Similar outcomes have been reported by Kubasiewicz et al. (2017), who demonstrated that HANP, either alone or in combination with other biomaterials, significantly enhances bone regeneration compared to untreated defects [56].

Importantly, no statistically significant differences were observed between HANP-modified mineral trioxide aggregate (MTA) and bioceramic (BC) groups in terms of new bone formation over time. This may be attributed to the similar physicochemical and biological properties of calcium silicate-based materials. Both MTA and BC release calcium and hydroxyl ions during hydration, creating an alkaline environment that promotes hydroxyapatite formation, mineralization, and tissue regeneration [57]. Their osteogenic potential has also been linked to their ability to stimulate angiogenesis and support osteoblastic differentiation through the release of bioactive ions [58]. Consistent with these findings, Rifaey et al. (2016) reported that bioceramic materials enhance osteoblastic differentiation in three-dimensional culture systems [59], while multiple studies have confirmed the capacity of MTA to promote new bone formation, as evidenced by active osteoblasts, collagen deposition, and mineralized matrix formation at defect sites [60,61].

For enhanced histological evaluation, Masson’s trichrome staining was employed to assess both the extent of newly formed bone and the quality of its mineralization across the experimental groups. This approach is widely used in bone defect studies to differentiate between immature fibrous tissue and mature mineralized bone, thereby providing insight into the progression of tissue repair [62].

The findings of the present study demonstrated a clear concentration-dependent effect of nano-hydroxyapatite (HANP) on bone regeneration, with the 4% HANP-modified mineral trioxide aggregate (MTA) and bioceramic (BC) groups showing optimal outcomes. This observation is consistent with previous reports indicating that HANP, when homogeneously distributed within a scaffold or matrix, closely mimics the mineral phase of native bone. Such biomimetic properties enhance osteogenic cell adhesion, proliferation, and subsequent matrix mineralization. The increased collagen deposition and mineralized matrix observed in Masson’s trichrome-stained sections further support the role of HANP in promoting osteogenic differentiation and extracellular matrix (ECM) maturation [30].

At the early healing stage (2 weeks), the control group predominantly exhibited immature, reticular collagen, whereas the 4% HANP groups demonstrated more organized collagen architecture with higher semiquantitative scores. This early enhancement in collagen organization may reflect accelerated formation of a provisional matrix conducive to osteoid deposition and subsequent remodeling. The bioactivity of calcium silicate-based materials, including their ability to release calcium ions, elevate local pH, and form an apatite-like layer, may further contribute to early extracellular matrix organization and osteogenic cellular responses, particularly in the 4% HANP groups [63].

By 4 weeks, collagen fiber deposition increased across most groups, with the 4% HANP + MTA group exhibiting the highest mean values and significant differences compared to the control and 6% HANP + BC groups. This trend aligns with previous in vitro findings demonstrating that calcium silicate-based materials support sustained matrix maturation through continuous ion release and surface mineral deposition, thereby promoting osteoblast differentiation and matrix formation [64].

At 8 weeks, most treated groups exhibited predominantly dense and well-organized collagen bundles, indicative of advanced extracellular matrix maturation. The 4% HANP groups continued to show superior outcomes, with significant differences compared to the control group, and the 4% HANP + MTA group demonstrated enhanced collagen deposition relative to the 6% HANP + BC group. This progressive organization reflects the natural remodeling process of bone healing, in which immature collagen is gradually replaced by thicker, more aligned fibers that serve as a scaffold for mineral deposition and tissue consolidation. These findings are consistent with previous studies linking increased collagen organization at later stages with enhanced matrix maturation and bone regeneration [65,66].

The superior collagen deposition and organization observed in the 4% HANP groups, compared to both 2% and 6% concentrations, likely reflect an optimal balance between nanoparticle availability and biological activity. At lower concentrations (2%), insufficient nanoparticle surface area may limit protein adsorption, cell attachment, and nucleation sites for apatite formation, resulting in only modest improvements over control conditions [67]. Conversely, higher concentrations (6%) may lead to nanoparticle agglomeration, reducing effective surface area and altering cell–material interactions, thereby diminishing biological performance. This phenomenon has been well documented for hydroxyapatite nanoparticles, which tend to aggregate under certain conditions, ultimately affecting their regenerative potential [68].

Taken together, the histological, histomorphometric, and Masson’s trichrome findings of the present study demonstrate that nano-hydroxyapatite (HANP)-modified calcium silicate-based materials enhance bone regeneration in maxillary bone defects in a concentration-dependent manner. Among the tested formulations, the 4% HANP groups consistently exhibited the most favorable outcomes, characterized by reduced inflammatory response, enhanced collagen organization, and increased new bone formation. These findings highlight the interplay between inflammation modulation and osteogenesis during the healing process and underscore the importance of optimizing nanoparticle concentration to achieve a balance between bioactivity and biocompatibility.

The results further suggest that HANP incorporation not only promotes osteoconduction but may also regulate the local biological environment to favor tissue regeneration. Future studies should incorporate histomorphometric measurements of bone area, as well as advanced three-dimensional imaging techniques, such as micro-computed tomography (micro-CT), to enable more precise quantification of bone volume and microarchitecture. In addition, molecular and immunohistochemical analyses targeting inflammatory mediators, macrophage polarization, and osteogenic markers would provide deeper insight into the mechanisms underlying the observed effects. Another limitation of this study is the absence of a 6-week observation point, which may have reduced the temporal resolution of the healing process between the 4- and 8-week evaluations. Future studies should include additional intermediate time points to better characterize bone regeneration. Longer observation periods are also recommended to evaluate the long-term maturation, remodeling, and functional stability of the regenerated bone.

## 5. Conclusions

Within the limitations of the present study, the incorporation of nano-hydroxyapatite enhanced the biological performance of calcium silicate-based sealers in a concentration-dependent manner. The 4% HANP formulation demonstrated the most favorable biological response among the concentrations evaluated, characterized by reduced inflammatory response, increased new bone formation, and improved collagen maturation. No statistically significant differences were observed between mineral trioxide aggregate (MTA) and bioceramic (BC) sealers at equivalent HANP concentrations, indicating comparable biological behavior. Collectively, these findings suggest that 4% HANP-modified calcium silicate sealers may represent promising biomaterials for bone regeneration and support their potential application in regenerative bone repair.

## Figures and Tables

**Table 1:** Inflammatory response to different concentrations of nano- hydroxyapatite (HANP) incorporated into MTA and bioceramic sealers at 2, 4, and 8 weeks.

Group	2 Weeks		4 Weeks		8 Weeks	
	(Mean ± SD)	(Median [IQR])	(Mean ± SD)	(Median [IQR])	(Mean ± SD)	(Median [IQR])
Control	1.6 ± 0.54 ^a^	2 [1–2]	1.4 ± 0.54 ^ab^	1 [1–2]	1.4 ± 0.54 ^a^	1 [1–2]
2% HANP + MTA	2.2 ± 0.44 ^a^	2 [2–2]	2.2 ± 0.44 ^a^	2 [2–2]	1.6 ± 0.54 ^a^	2 [1–2]
2% HANP + BC	2.2 ± 0.44 ^a^	2 [2–2]	1.6 ± 0.89 ^a^	1 [1–2]	1.8 ± 0.83 ^a^	2 [1–2]
4% HANP + MTA	1.2 ± 0.44 ^a^	1 [1–1]	0.6 ± 0.54 ^b^	1 [0–1]	0.4 ± 0.54 ^a^	0 [0–1]
4% HANP + BC	1.4 ± 0.54 ^a^	1 [1–2]	0.6 ± 0.54 ^b^	1 [0–1]	0.6 ± 0.54 ^a^	1 [0–1]
6% HANP + MTA	2.2 ± 0.44 ^a^	2 [2–2]	1.4 ± 0.54 ^ab^	1 [1–2]	1.4 ± 0.54 ^a^	1 [1–2]
6% HANP + BC	2.0 ± 0.70 ^a^	2 [1–2]	1.4 ± 0.89 ^ab^	1 [1–1]	1.6 ± 0.54 ^a^	2 [1–2]

(SD): standard deviation. Different superscript letters (a, b, ab) within the same column indicate statistically significant differences among groups (*p* < 0.05); values sharing at least one letter are not significantly different (*p* ≥ 0.05).

**Table 2:** New bone formation scores in untreated empty control defects and HANP-modified MTA and BC groups at 2, 4, and 8 weeks.

Group	2 Weeks		4 Weeks		8 Weeks	
	(Mean ± SD)	(Median [IQR])	(Mean ± SD)	(Median [IQR])	(Mean ± SD)	(Median [IQR])
Control	1.2 ± 0.44 ^ab^	1 [1–1]	1.0 ± 0.70 ^ab^	1 [1–1]	1.4 ± 0.54 ^ab^	1 [1–2]
2% HANP + MTA	1.0 ± 0.00 ^ab^	1 [1–1]	1.2 ± 0.83 ^ab^	1 [1–2]	1.4 ± 0.54 ^ab^	1 [1–2]
2% HANP + BC	0.8 ± 0.44 ^b^	1 [1–1]	1.0 ± 0.00 ^b^	1 [1–1]	1.2 ± 0.44 ^b^	1 [1–1]
4% HANP + MTA	2.2 ± 0.44 ^a^	2 [2–2]	2.6 ± 0.54 ^a^	3 [2–3]	2.6 ± 0.54 ^a^	3 [2–3]
4% HANP + BC	2.0 ± 0.70 ^ab^	2 [2–2]	2.4 ± 0.54 ^ab^	2 [2–3]	2.4 ± 0.54 ^ab^	2 [2–3]
6% HANP + MTA	1.6 ± 0.54 ^ab^	2 [1–2]	2.0 ± 0.70 ^ab^	2 [2–2]	1.8 ± 0.44 ^ab^	2 [2–2]
6% HANP + BC	1.4 ± 0.54 ^ab^	1 [1–2]	1.4 ± 0.54 ^ab^	1 [1–2]	1.6 ± 0.54 ^ab^	2 [1–2]

(SD): standard deviation. Different superscript letters (a, b, ab) within the same column indicate statistically significant differences among groups (*p* < 0.05); values sharing at least one letter are not significantly different (*p* ≥ 0.05).

**Table 3:** Collagen fiber deposition scores (mean ± standard deviation and median [interquartile range]) in untreated empty control defects, and nano- hydroxyapatite (HANP)-modified MTA and bioceramic (BC) groups at 2, 4, and 8 weeks.

Group	2 Weeks		4 Weeks		8 Weeks	
	(Mean ± SD)	(Median [IQR])	(Mean ± SD)	(Median [IQR])	(Mean ± SD)	(Median [IQR])
Control	0.00 ± 0.00 ^a^	0 [0–0]	0.40 ± 0.55 ^a^	0 [0–1]	1.00 ± 0.00 ^a^	1 [1–1]
2% HANP + MTA	0.40 ± 0.55 ^ab^	0 [0–1]	0.80 ± 0.45 ^ab^	1 [1–1]	1.40 ± 0.55 ^ab^	1 [1–2]
2% HANP + BC	0.20 ± 0.45 ^a^	0 [0–0]	0.60 ± 0.55 ^ab^	1 [0–1]	1.00 ± 0.00 ^a^	1 [1–1]
4% HANP + MTA	1.00 ± 0.00 ^c^	1 [1–1]	1.40 ± 0.55 ^b^	1 [1–2]	2.00 ± 0.00 ^c^	2 [2–2]
4% HANP + BC	1.00 ± 0.00 ^ab^	1 [1–1]	1.20 ± 0.45 ^b^	1 [1–1]	1.50 ± 0.55 ^ab^	2 [1–2]
6% HANP + MTA	0.00 ± 0.00 ^a^	0 [0–0]	1.00 ± 0.00 ^ab^	1 [1–1]	1.40 ± 0.55 ^ab^	1 [1–2]
6% HANP + BC	0.60 ± 0.55 ^ab^	1 [0–1]	0.40 ± 0.55 ^a^	0 [0–1]	1.20 ± 0.45 ^ab^	1 [1–1]

(SD): standard deviation. Different superscript letters (a, b, c, ab) within the same column indicate statistically significant differences among groups (*p* < 0.05); values sharing at least one letter are not significantly different (*p* ≥ 0.05).

## Data Availability

The datasets generated and analyzed during the present study, including histological images and histomorphometric measurements, are available from the corresponding author upon reasonable request.

## Abbreviations

The following abbreviations are used in this manuscript:

BC	Bioceramic sealer
BM	Body weight
DPX	Dibutyl phthalate polystyrene xylene
ECM	Extracellular matrix
HANP	Nano-hydroxyapatite
IL-1	Interleukin-1
IL-6	Interleukin-6
M1	Pro-inflammatory macrophages
M2	Anti-inflammatory macrophages
MT	Masson’s trichrome
MTA	Mineral trioxide aggregate
RunX2	Runt-related transcription factor 2
SD	Standard deviation
TGF-β	Transforming growth factor beta
